# Supplementary material for: Meta-Analysis and Gene Set Enrichment Relative to ER Status Reveal Elevated Activity of MYC and E2F in the “Basal” Breast Cancer Subgroup
Source: PLoS One. 2009 Mar 9;4(3):e4710. doi: 10.1371/journal.pone.0004710 (PMC2650420; doi:10.1371/journal.pone.0004710)
Supplement: Dataset S1 — GSEA results from first screen. The file contains a zipped archive of the HTML results of the second GSEA screen. Reports for enriched or depleted datasets may be accessed from the “index.html” link within the my_analysis.GseaPreranked.1219975950625.rpt.zip ZIP archive. Enrichment plots are available for each of the 50 most enriched and depleted gene sets. (13.32 MB ZIP) [file pone.0004710.s001.zip › my_analysis.GseaPreranked.1219975950625.rpt.zip!//BRCA_PROGNOSIS_NEG.html]

Details for gene set BRCA\_PROGNOSIS\_NEG[GSEA]

|  || Dataset | zSco.ERneg.to.ERpos\_collapsed |
| Phenotype | NoPhenotypeAvailable |
| Upregulated in class | na\_pos |
| GeneSet | BRCA\_PROGNOSIS\_NEG |
| Enrichment Score (ES) | 0.72650117 |
| Normalized Enrichment Score (NES) | 2.9657886 |
| Nominal p-value | 0.0 |
| FDR q-value | 0.0 |
| FWER p-Value | 0.0 |
Table: GSEA Results Summary

  

Fig 1: Enrichment plot: BRCA\_PROGNOSIS\_NEG      
 Profile of the Running ES Score & Positions of GeneSet Members on the Rank Ordered List

  

| PROBE | GENE SYMBOL | GENE\_TITLE | RANK IN GENE LIST | RANK METRIC SCORE | RUNNING ES | CORE ENRICHMENT || 1 | CTSL2 |  |  | 1 | 7.773 | 0.0238 | Yes |
| 2 | ORC6L |  |  | 33 | 6.507 | 0.0415 | Yes |
| 3 | BUB1 |  |  | 41 | 6.445 | 0.0608 | Yes |
| 4 | CENPA |  |  | 51 | 6.315 | 0.0795 | Yes |
| 5 | DKFZP762E1312 |  |  | 61 | 6.161 | 0.0978 | Yes |
| 6 | NMB |  |  | 71 | 6.063 | 0.1158 | Yes |
| 7 | TRIP13 |  |  | 96 | 5.890 | 0.1321 | Yes |
| 8 | LRP12 |  |  | 109 | 5.815 | 0.1491 | Yes |
| 9 | RRAGD |  |  | 122 | 5.723 | 0.1658 | Yes |
| 10 | CTPS |  |  | 123 | 5.706 | 0.1833 | Yes |
| 11 | FAM64A |  |  | 137 | 5.658 | 0.1997 | Yes |
| 12 | GGH |  |  | 161 | 5.504 | 0.2149 | Yes |
| 13 | IVNS1ABP |  |  | 172 | 5.467 | 0.2310 | Yes |
| 14 | MELK |  |  | 181 | 5.424 | 0.2471 | Yes |
| 15 | MCM6 |  |  | 198 | 5.317 | 0.2622 | Yes |
| 16 | KIF14 |  |  | 221 | 5.215 | 0.2766 | Yes |
| 17 | CKS2 |  |  | 239 | 5.145 | 0.2911 | Yes |
| 18 | KNTC2 |  |  | 256 | 5.074 | 0.3055 | Yes |
| 19 | TFRC |  |  | 259 | 5.071 | 0.3210 | Yes |
| 20 | OXCT1 |  |  | 283 | 4.991 | 0.3346 | Yes |
| 21 | GPSM2 |  |  | 291 | 4.963 | 0.3493 | Yes |
| 22 | STMN1 |  |  | 310 | 4.907 | 0.3631 | Yes |
| 23 | EZH2 |  |  | 312 | 4.888 | 0.3780 | Yes |
| 24 | MCCC1 |  |  | 326 | 4.861 | 0.3920 | Yes |
| 25 | CDC25B |  |  | 340 | 4.826 | 0.4059 | Yes |
| 26 | CENPN |  |  | 352 | 4.783 | 0.4198 | Yes |
| 27 | ASPM |  |  | 359 | 4.767 | 0.4340 | Yes |
| 28 | RFC4 |  |  | 370 | 4.741 | 0.4478 | Yes |
| 29 | SYNCRIP |  |  | 402 | 4.679 | 0.4598 | Yes |
| 30 | DEPDC1 |  |  | 406 | 4.663 | 0.4740 | Yes |
| 31 | CCNB2 |  |  | 431 | 4.589 | 0.4863 | Yes |
| 32 | C16ORF61 |  |  | 447 | 4.520 | 0.4990 | Yes |
| 33 | SMC4 |  |  | 448 | 4.519 | 0.5129 | Yes |
| 34 | AURKA |  |  | 460 | 4.483 | 0.5259 | Yes |
| 35 | MAD2L1 |  |  | 477 | 4.446 | 0.5384 | Yes |
| 36 | MAPRE2 |  |  | 507 | 4.333 | 0.5495 | Yes |
| 37 | NMU |  |  | 520 | 4.311 | 0.5618 | Yes |
| 38 | DLG7 |  |  | 521 | 4.306 | 0.5751 | Yes |
| 39 | PRC1 |  |  | 543 | 4.253 | 0.5866 | Yes |
| 40 | SPBC25 |  |  | 606 | 4.126 | 0.5946 | Yes |
| 41 | ASNS |  |  | 618 | 4.099 | 0.6064 | Yes |
| 42 | EXT1 |  |  | 673 | 3.986 | 0.6145 | Yes |
| 43 | PCTK1 |  |  | 708 | 3.915 | 0.6240 | Yes |
| 44 | HRB |  |  | 752 | 3.840 | 0.6326 | Yes |
| 45 | PIR |  |  | 757 | 3.834 | 0.6441 | Yes |
| 46 | UCHL5 |  |  | 765 | 3.814 | 0.6553 | Yes |
| 47 | SLC7A1 |  |  | 798 | 3.782 | 0.6645 | Yes |
| 48 | CA9 |  |  | 909 | 3.623 | 0.6673 | Yes |
| 49 | MTMR2 |  |  | 965 | 3.531 | 0.6740 | Yes |
| 50 | SACS |  |  | 1039 | 3.426 | 0.6790 | Yes |
| 51 | ADM |  |  | 1046 | 3.419 | 0.6891 | Yes |
| 52 | COL4A2 |  |  | 1108 | 3.351 | 0.6948 | Yes |
| 53 | HMGB3 |  |  | 1128 | 3.330 | 0.7036 | Yes |
| 54 | PFKP |  |  | 1165 | 3.281 | 0.7110 | Yes |
| 55 | FBXO5 |  |  | 1304 | 3.137 | 0.7102 | Yes |
| 56 | FLT1 |  |  | 1486 | 2.957 | 0.7056 | Yes |
| 57 | ATAD2 |  |  | 1532 | 2.917 | 0.7112 | Yes |
| 58 | GMPS |  |  | 1543 | 2.907 | 0.7194 | Yes |
| 59 | PITRM1 |  |  | 1607 | 2.843 | 0.7233 | Yes |
| 60 | PTDSS1 |  |  | 1806 | 2.686 | 0.7166 | Yes |
| 61 | TSPYL5 |  |  | 1866 | 2.634 | 0.7203 | Yes |
| 62 | MMP9 |  |  | 1891 | 2.616 | 0.7265 | Yes |
| 63 | ECT2 |  |  | 2026 | 2.509 | 0.7241 | No |
| 64 | C1ORF106 |  |  | 2166 | 2.409 | 0.7210 | No |
| 65 | ESM1 |  |  | 2326 | 2.308 | 0.7161 | No |
| 66 | NDRG1 |  |  | 2463 | 2.218 | 0.7126 | No |
| 67 | BNIP3 |  |  | 2662 | 2.111 | 0.7041 | No |
| 68 | PSMD2 |  |  | 2759 | 2.063 | 0.7032 | No |
| 69 | PAQR3 |  |  | 2863 | 2.005 | 0.7016 | No |
| 70 | TMEM45A |  |  | 3492 | 1.713 | 0.6594 | No |
| 71 | C20ORF46 |  |  | 3525 | 1.696 | 0.6622 | No |
| 72 | RAD21 |  |  | 3642 | 1.642 | 0.6585 | No |
| 73 | AYTL2 |  |  | 3655 | 1.639 | 0.6626 | No |
| 74 | VEGF |  |  | 3911 | 1.549 | 0.6481 | No |
| 75 | PRAME |  |  | 3945 | 1.537 | 0.6503 | No |
| 76 | PLEKHA1 |  |  | 4137 | 1.457 | 0.6404 | No |
| 77 | CDC42BPA |  |  | 4330 | 1.379 | 0.6301 | No |
| 78 | ARMC1 |  |  | 4439 | 1.341 | 0.6261 | No |
| 79 | DTL |  |  | 4509 | 1.314 | 0.6249 | No |
| 80 | INTS7 |  |  | 4743 | 1.239 | 0.6111 | No |
| 81 | GNAZ |  |  | 4789 | 1.226 | 0.6114 | No |
| 82 | GPR126 |  |  | 4994 | 1.158 | 0.5996 | No |
| 83 | GBE1 |  |  | 5011 | 1.151 | 0.6019 | No |
| 84 | PTPLB |  |  | 5075 | 1.125 | 0.6006 | No |
| 85 | PGK1 |  |  | 5836 | 0.865 | 0.5458 | No |
| 86 | PSMD7 |  |  | 6263 | 0.727 | 0.5158 | No |
| 87 | DEGS1 |  |  | 6528 | 0.640 | 0.4979 | No |
| 88 | MTDH |  |  | 6928 | 0.510 | 0.4693 | No |
| 89 | STK3 |  |  | 7585 | 0.275 | 0.4205 | No |
| 90 | DIAPH3 |  |  | 7628 | 0.259 | 0.4181 | No |
| 91 | MRPL13 |  |  | 8230 | 0.023 | 0.3728 | No |
| 92 | DCK |  |  | 8935 | -0.280 | 0.3204 | No |
| 93 | MGAT4A |  |  | 10559 | -1.215 | 0.2014 | No |
| 94 | IGFBP5 |  |  | 11829 | -2.352 | 0.1127 | No |
Table: GSEA details [plain text format]

  

Fig 2: BRCA\_PROGNOSIS\_NEG: Random ES distribution      
 Gene set null distribution of ES for **BRCA\_PROGNOSIS\_NEG**
